# Reporting the whole story: Analysis of the ‘out‐of‐scope’ questions from the James Lind Alliance Teenage and Young Adult Cancer Priority Setting Partnership Survey

**DOI:** 10.1111/hex.13276

**Published:** 2021-07-10

**Authors:** Faith Gibson, Lorna A. Fern, Bob Phillips, Helen Gravestock, Sonia Malik, Amy Callaghan, Karen Dyker, Mike Groszmann, Leila Hamrang, Rachael Hough, Demi McGeachy, Sue Morgan, Sam Smith, Sheela Upadhyaya, Helen Veitch, Max Williamson, Jeremy Whelan, Susie Aldiss

**Affiliations:** ^1^ Centre for Outcomes and Experience Research in Children's Health, Illness and Disability Great Ormond Street Hospital for Children NHS Foundation Trust London UK; ^2^ School of Health Sciences University of Surrey Guildford UK; ^3^ Cancer Clinical Trials Unit University College London Hospitals NHS Foundation Trust London UK; ^4^ Centre for Reviews and Dissemination University of York York UK; ^5^ Department of Paediatric Haematology and Oncology Leeds Children's Hospital Leeds UK; ^6^ Policy, Influencing and Voice Young Lives vs Cancer London UK; ^7^ Policy and Influencing Young Lives vs Cancer London UK; ^8^ Teenage and Young Adult Cancer Priority Setting Partnership steering group Glasgow UK; ^9^ Clinical Oncology Department Clinical Oncology Department St James's University Hospital Leeds UK; ^10^ Psychological Medicine Department University College London Hospitals NHS Foundation Trust London UK; ^11^ Teenage and Young Adult Cancer Priority Setting Partnership steering group Manchester UK; ^12^ Department of Adolescent Haematology, Children and Young People's Cancer Service University College London Hospitals NHS Foundation Trust London UK; ^13^ Teenage and Young Adult Cancer Service Ward L33 Leeds General Infirmary Leeds Teaching Hospitals NHS Trust Leeds UK; ^14^ Teenage Cancer Trust London UK; ^15^ Trials and Studies Coordinating Centre The James Lind Alliance National Institute for Health Research Evaluation University of Southampton Southampton UK; ^16^ Teenage and Young Adult Cancer Priority Setting Partnership steering group London UK; ^17^ Division of Oncology University College London Hospitals NHS Foundation Trust London UK

**Keywords:** cancer, information, James Lind Alliance, support, teenage, young adult

## Abstract

**Objective:**

We conducted a UK‐wide survey to identify the top 10 research questions for young people's cancer. We conducted secondary analysis of questions submitted, which were ‘out‐of‐scope’ of the original survey aim. We sought to disseminate these questions, to inform practice, policy and the development of potential interventions to support young people with cancer.

**Design:**

James Lind Alliance Priority Setting Partnership.

**Participants:**

Young people aged 13‐24 with a current/previous cancer diagnosis, their families/friends/partners and professionals who work with this population.

**Methods:**

Eight hundred and fifty‐five potential research questions were submitted, and 326 were classified as ‘out‐of‐scope’. These questions, along with 49 ‘free‐text’ comments, were analysed using thematic analysis.

**Results:**

The 375 out‐of‐scope questions and comments were submitted by: 68 young people, 81 family members/partners/friends and 42 professionals. Ten overarching themes were identified: diagnostic experience; communication; coordination of care; information needs and lack of information; service provision; long‐term effects and aftercare support; family support; financial impact; end‐of life care; and research methods and current research.

**Conclusions:**

The need to tailor services, information and communication is a striking thread evidenced across the ‘out‐of‐scope’ questions. Gaps in information highlight implications for practice in revisiting information needs throughout the cancer trajectory. We must advocate for specialist care for young people and promote the research priorities and these findings to funding bodies, charities, young people and health and social care policymakers, in order to generate an evidence base to inform effective interventions across the cancer trajectory and improve outcomes.

**Patient/public contributions:**

Patients and carers were equal stakeholders throughout.

## 1 INTRODUCTION

Teenagers and young adults (TYA) with cancer require specialist care to meet their unique physical, psychological and social needs.^1^ They present with a spectrum of cancer types requiring complex care involving many stakeholders.^2^ In the United Kingdom (UK), care is delivered in National Health Service (NHS) age‐appropriate specialist TYA cancer centres and ‘designated’ hospitals, additional resource is provided by the third (charitable) sector. For TYA services to deliver patient‐centred care meeting the needs of this distinct cancer population, the evidence base provided by research must reflect service users' needs and concerns.^3^ It is increasingly recognized that research priorities between clinicians, researchers and patient/public populations can differ. James Lind Alliance (JLA) Research Priority Setting Partnerships (PSP) aim to identify research priorities involving all stakeholders as equal partners.^1^ The process involves patients, members of the public and professionals submitting their unanswered research questions.^4^ Invariably, each PSP will receive submissions that are not questions, but are more akin to personal statements, and also where a research question is not readily identifiable or not within remit of that particular PSP, these are classified as ‘out‐of‐scope’ questions.^4^

**Figure 1:**
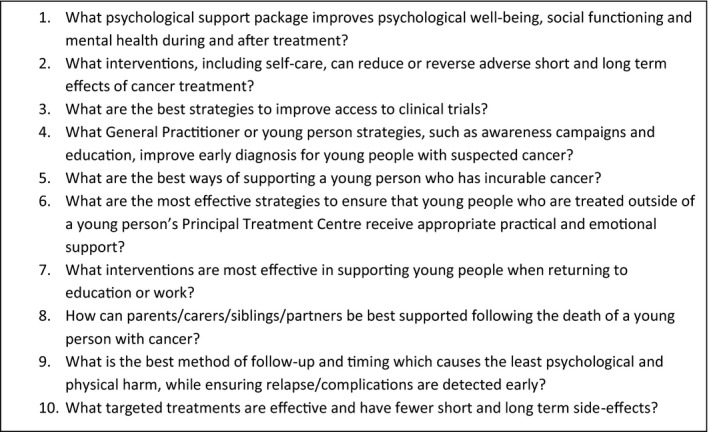
Top 10 research priorities for teenage and young adult cancer

The Teenage and Young Adult Cancer JLA PSP identified the top 10 research questions for young people with cancer (Figure 1), aged 13‐24, bringing together young people, carers and multidisciplinary professionals.^5^ The JLA principles are transparency, inclusivity and avoiding research waste, and researchers are advised to have a strategy for ‘out‐of‐scope’ questions prior to starting the JLA process.^6^, ^7^ Many opt to transfer the data onto organizations who can utilize the information, for example relevant charities and social research institutions.^4^ We identified only one other PSP who analysed the subject content of their ‘out‐of‐scope’ questions and produced a report.^8^ The TYA Cancer PSP generated over 800 potential research questions, many of which were ‘out‐of‐scope’. The ‘out‐of‐scope’ questions tended to focus on personal experiences, questions about information and support, and other related concerns and uncertainties about cancer and service provision for young people. Some respondents shared their experiences in a narrative form, focusing on sharing what had gone wrong and/or how services could be delivered. In keeping with the JLA principles of avoiding research waste, we present a supplementary analysis of the TYA Cancer PSP ‘out‐of‐scope’ questions ensuring the voices, and concerns, of all people who took part in the survey have the opportunity to be heard.

This secondary analysis aimed to explore the ‘out‐of‐scope’ questions, how they can inform practice and policy and influence the development of future interventions to support young people with cancer.

## 2 METHODS

We followed JLA methodology^4^; previously published.^4^, ^5^ Briefly, an expert steering group including young people and professionals oversaw the project, approved aims/objectives and survey materials, and cleaned data. Our aim was, ‘To identify gaps and unanswered questions in research, the answers to which may reduce the individual and societal burden of young people's cancer’.^5^ The scope was broad including the following: prevention; causes; diagnosis; treatment; care; follow‐up; survivorship; relapse; and end‐of‐life care, reflecting the cancer timeline. The online UK‐wide survey ran between October and December 2016 gathering unanswered questions and comments related to young people's cancer. Participants were diagnosed with cancer between 13 and 24 years, and relatives/friends/partners/carers and professionals working with young people.

### 2.1 Data analysis

**Figure 2:**
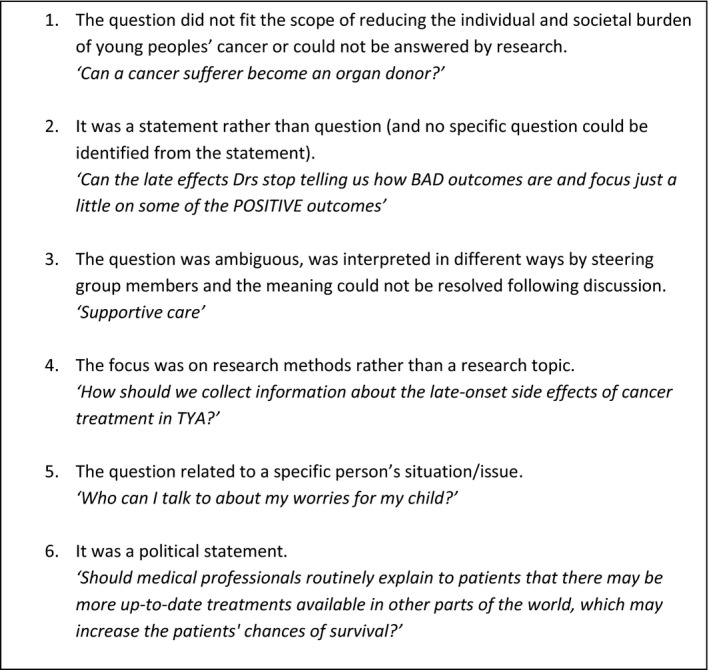
Initial out‐of‐scope question categories and examples

This involved three stages. During the first stage, all questions submitted to the survey were examined and free‐text sections studied for further questions.^4^, ^5^ Three project coordinators (FG/LF/SA) sorted the questions into themes to ease review and discussion. During this sorting, out‐of‐scope questions were identified. Out‐of‐scope questions were those that did not fit our project scope (‘*To identify gaps and unanswered questions in research, the answers to which may reduce the individual and societal burden of young people's* *cancer’*) or were unanswerable by research. The JLA guidebook offers the following guidance ‘*Some responses may not need to be answered research, for example, they may be questions seeking further information or advice on a topic, or issues around awareness*.’(p.43).^4^ Identifying out‐of‐scope questions was an iterative process, checked and agreed by the steering group. Out‐of‐scope questions were discussed at steering group meetings. The steering group also reviewed the full list of submitted questions to identify any further out‐of‐scope questions. Where the steering group could not reach a consensus on whether an entry was out‐of‐scope, the young people made the final decision as to whether an entry could be interpreted as a research question or was ‘out‐of‐scope’. Six broad categories of out‐of‐scope questions were identified (Figure 2). In the second stage, out‐of‐scope questions were analysed using thematic analysis by two steering group members (SM/HG) and three project coordinators, working in teams (FG/SA/LF).^3^ Responses were coded by two with checking and further refinement by three members (FG/SA/LF). This process involved the following: (a) familiarization with all questions; (b) developing a series of categories; (c) grouping similar questions within categories; (d) checking groupings, combining into overarching themes; and (e) discussion and refinement of themes to ensure agreement. The final stage involved three team members (LF/FG/SA), who discussed the themes and identified potential interventions. Discussion was expanded to include reflections on the top 10 priority list. At this point, we were also able to map our themes to the top 10 list. These were then supplemented and approved by the wider steering group, which includes representatives from: the third sector; teenage and young adult cancer nursing; policy; clinicians from haematology, paediatrics, and adult oncology; and psychology and youth support.

## 3 RESULTS

Of 855 questions submitted by 292 participants, 326 were classified ‘out‐of‐scope’. Forty‐nine free‐text comments were also included in our analysis. Questions/comments were submitted by 191 participants: 68 (36%) young people; 81 (42%) family members/partners/friends; and 42 (22%) professionals. The majority of family members/partners/friends were parents/carers (n = 59; 73%); 11 (14%) were friends; nine (11%) were relatives; and two were partners (2%). A range of professionals submitted out‐of‐scope questions/comments: 13 (31%) doctors; 12 (29%) nurses; nine (21%) allied health professionals; and eight (19%) ‘Other’. ‘Other' included third‐sector professionals and academic researchers. Participant demographics are shown in Appendix S1.

Ten overarching themes were identified: diagnostic experience; communication; coordination of care; information needs and lack of information; service provision; long‐term effects and aftercare support; family support; financial impact; end‐of‐life care; and research methods and current research. We report these with illustrative verbatim quotes from participants: typographical errors remain. We then propose a list of potential interventions in Table 1, aligned with the top 10 priorities, to situate our secondary analysis, within the context of the ‘whole story’.

**Table 1:** Overarching themes, subthemes, potential interventions and the relevant top 10 question, many of which would need to be answered to inform the intervention

Theme	Subthemes	Potential interventions	Relevant top 10 question
Diagnostic experience	Low awareness of cancer in young people Low awareness of potential cancer symptoms in young people Communication between GPs, young people and families	Professional and public awareness campaigns on cancer in young people Teenage and young adult‐friendly primary care services	No.4 What general practitioner or young person strategies, such as awareness campaigns and education, improve early diagnosis for young people with suspected cancer?
Communication	Timely communication Communicating difficult issues Communicating choice and young peoples role in decisions made Communication with health‐care providers Improving communication Meeting communication needs of the family Open and honest communication Communication with employers Communication with friends	Revisit important information and check understanding. Ensure all professionals in young person's service have advanced communication training Routinely arrange part of the appointment with young people on their own to allow young people space to ask personal/sensitive questions. Routinely arrange part of the appointment with parents on their own to allow the opportunity to ask personal/sensitive questions (which could be answered without breaching confidentiality) Key worker to liaise between family and employer Educate young people on how to talk to friends about cancer Raise public awareness of cancer in young people	No. 6 What are the most effective strategies to ensure that young people who are treated outside of a young person's Principal Treatment Centre receive appropriate practical and emotional support? No.5 What are the best ways of supporting a young person who has incurable cancer? No.7 What interventions are most effective in supporting young people when returning to education or work?
Coordination of care	Transition, active‐palliative care With primary care Beyond 25 years Across physical rehabilitation, psychosocial support, social care and education	Key worker to liaise between services and families who has oversight of the young person's treatment, care, social and educational/employer needs Clear guidance of who to contact and for what within each service Transition service for those leaving TYA care into adult services. Link between secondary care and primary care throughout treatment and afterwards to inform primary care what cancer/treatment‐related symptoms to look for and what short‐term and late effects may occur and where to refer to.	No.6 What are the most effective strategies to ensure that young people who are treated outside of a young person's Principal Treatment Centre receive appropriate practical and emotional support?
Information needs and lack of information	Cause of cancer, how it grows Prognosis Treatment Access to treatment Side‐effects Available services Provision of support (psychosocial/practical) Relapse Fertility Long‐term effects What can I do to help myself? Treatment outside of the UK	Age‐appropriate information for young people and their families across the cancer timeline. Research to fill the information gaps where there is little or no evidence base. Dissemination of research results to health‐care professionals, young people and their families. Revisiting information needs with young people and their families throughout the cancer timeline.	No. 1 What psychological support package improves psychological well‐being, social functioning and mental health during and after treatment? No. 2 What interventions, including self‐care, can reduce or reverse adverse short‐ and long‐term effects of cancer treatment?
Service provision	Age‐appropriate care Access to treatment Provision of support (psychosocial/practical) Relapse Rehabilitation Access to complementary therapies Holistic care Peer group support Long‐term effects	Equitable access to age‐appropriate services Access to new drugs and clinical trials in TYA specialist centres Good communication and links between specialist TYA centres and local hospitals Information of peer‐to‐peer groups and support events Clear pathways from active treatment to follow‐up Research to underpin evidence of complementary therapies	No.1 What psychological support package improves psychological well‐being, social functioning and mental health during and after treatment? No.2 What interventions, including self‐care, can reduce or reverse adverse short‐ and long‐term effects of cancer treatment? No.3 What are the best strategies to improve access to clinical trials? No.6 What are the most effective strategies to ensure that young people who are treated outside of a young person's Principal Treatment Centre receive appropriate practical and emotional support? No. 10 What targeted treatments are effective and have fewer short‐ and long‐term side‐effects?
Long‐term effects and aftercare support	Feeling abandoned Fertility Provision of support (psychosocial/practical) Long‐term follow‐up positive/negative On‐going surveillance‐positive/negative maintaining life goals	Clear transition pathways from active treatment to follow‐up care Support for parents following end of treatment Age‐appropriate fertility services including information on how to access frozen sperm, eggs and embryos Evidence‐based follow‐up pathways Clear information for young people and their families on frequency of follow‐up Information on available support groups and peer‐to‐peer events On‐going psychosocial support to attain life goals	No. 1 What psychological support package improves psychological well‐being, social functioning and mental health during and after treatment? No. 2 What interventions, including self‐care, can reduce or reverse adverse short‐ and long‐term effects of cancer treatment? No. 7 What interventions are most effective in supporting young people when returning to education or work? No. 9 What is the best method of follow‐up and timing that causes the least psychological and physical harm, while ensuring relapse/complications are detected early?
Family support	Parental role Sibling support Practical and emotional support During remission At relapse Post‐death	Information about available support groups and peer‐to‐peer events for parents and siblings Contact with the family after treatment has ended Post‐death support services for families	No. 8 How can parents/carers/siblings/partners be best supported following the death of a young person with cancer?
Financial impact	Communicating with employers Communicating medical history What financial support is available Health insurance	Clear age‐appropriate information about financial support available to young people and their carers Information on employment rights for young people and their carers Education on attaining life goals following treatment Key worker to assist with identification of financial opportunities and liaise with employer	No. 7 What interventions are most effective in supporting young people when returning to education or work?
End‐of‐life care	Communication Family support Information needs and lack of information Provision of support (psychosocial/practical) Service provision	Palliative care services that are skilled in dealing with young people Support for the family and siblings Communication training for professionals	No. 5 What are the best ways of supporting a young person who has incurable cancer? No.6 What are the most effective strategies to ensure that young people who are treated outside of a young person's Principal Treatment Centre receive appropriate practical and emotional support? No. 8 How can parents/carers/siblings/partners be best supported following the death of a young person with cancer?
Research methods and current research	Research methodology Low‐priority area Feedback on research findings Current research topics	Increased funding for rare cancers Increasing funders awareness of the TYA JLA PSP Funders asking for plans for dissemination of results back to participants as part of funding submission Ensuring patient/public involvement is imbedded in research to facilitate information back to participants Public facing database of available research studies, along with results of closed studies	No.3 What are the best strategies to improve access to clinical trials?

### 3.1 Diagnostic experience

Many participants commented about the diagnostic experience, with young people describing not feeling listened to by their general practitioner (GP) leading to long periods between seeking help and diagnosis:

Doctors should listen to young people to get diagnosed quickly. (Patient/former patient)

This was echoed by parents:

My son died at the age of 23, 8 months after diagnosis. Despite the initial prognosis being good. However getting to the diagnosis in the first place was too slow. (Parent/carer)

Related to this, many participants asked about cancer awareness campaigns for cancer in young people:

What is being done to raise awareness of childhood cancer symptoms within the healthcare sector? (Parent/carer)

### 3.2 Communication

All participant groups submitted questions/comments relating to communication. Suggestions for how communication between families, professionals and friends might be improved were raised. Open, timely, honest communication in a way that met families' needs was clearly important:

From experience I found that the consultants and doctors were afraid to be completely open and frank with my daughter despite her 17 years. She felt that they kept things from her (Parent/carer)

Help, support, guide, but be truthful, honest and drop the sugar coating (Parent/carer)

There were questions about the best way for professionals to communicate with young people and their families, such as how best to provide results to families and preferred modes of communication. Information overload was an issue; strategies to overcome this were suggested, for example provision of written information alongside verbal information and revisiting information over time:

Why is not all the support care explained more than once? Eg CNS (Clinical Nurse Specialist) support. I didn't realise I had one for about 6 years post chronic myeloid leukaemia diagnosis (Patient/former patient)

The importance of offering the opportunity for parents and young people to speak with professionals on their own was raised by professionals and parents:

Youngsters are often embarrassed to ask the questions that are important to them anyway, irrespective of cancer… relationships, sex, jobs, leaving home. (Parent/carer)

It was difficult to ask questions in front of our 18 year old as due to his age he was considered an adult. We wanted to keep bad news away from him. We could never ask what were his chances and will it reoccur. For fear of scaring him. Once he was off the ward this was more difficult as we were always at consultations together. (Parent/carer)

Questions were raised about involving young people in decision making around their treatment options, around refusing treatments or suggesting their preferred treatment. Young people asked questions regarding the best way to communicate about their cancer with other people, including friends and employers.

### 3.3 Coordination of care

Participants commented on the need for more joined‐up and coordinated services. This included better coordination across different services such as education, health and social care, between active treatment and palliative care teams and between treatment centres and primary care. One young person described being, *‘sent up and down the country’*, with a lack of communication between the treatment centre and local services. Parents also commented on experiences of uncoordinated care:

The radiotherapy was exceptionally well organised, but the chemotherapy was very difficult. What can be done to support the chemo staff to be able to communicate better and make the process clearer for patients and carers? (Parent/carer)

Difficulties accessing health care following treatment were described:

GP must be better informed ours said I know nothing about brain tumours. If having seizures be referred to a specialist of brain tumour seizure. Again our surgery said to my son I cannot help you in that are, so who can? (Parent/Carer)

Once finishing treatment why do I seemed to get pushed between the hospital and my GP, as nobody seems to want to assist with non‐cancer related problems? (Patient/former patient)

### 3.4 Information needs and lack of information

Concerns were raised by all participant groups and spanned the cancer trajectory. Young people asked questions about the causes of cancer, how they developed it, whether they could have done anything to avoid getting it or do anything to stop it returning.

Young people and carers felt they did not feel informed about the short‐ and long‐term treatment side‐effects, wishing they had been forewarned about a wide range of adverse effects and optimal management strategies:

Why did you not inform me about the late effects I am now experiencing? (Patient/former patient)

Realistic information on fatigue for young people, with examples of how to manage fatigue in a young person's life as always found it was gear towards older patients the information on fatigue. (Partner)

My daughter was diagnosed with avascular necrosis due to the steroids for cancer treatment… why are parents not made more aware of this side effect? (Parent/carer)

What will my fertility options be for the future and where can I get the best support for fertility issues in my region? (Patient/former patient)

Young people wanted information on long‐term survivorship and lifestyle choices**:**

What sort of problems should I be particularly aware of as a survivor? (for instance, i know i am supposed to have the flu jab, is there anything else?) (Patient/former patient)

Parents/carers asked about relapse and survival. Participants asked where to receive information about treatments being received now and previously, options if current treatments failed and availability of alternative treatments including complementary therapies and overseas:

How serious was my leukaemia? I have no idea of its subtype! Is there any way that I could have a proper 'debrief' with regards my treatment? I know, 17 years later! (Patient/former patient)

### 3.5 Service provision

Service provision was a further focus, with psychosocial and practical support being the main component. Participants had questions about support during and after treatment, what was available locally and how to access it, as finding this information was difficult. In addition to formal psychological support and counselling, participants sought information on peer support and young people's groups/forums:

Is there an organisation that could pull together, and co‐ordinate various activity and social inclusion groups to make it easier for young persons with or who have had cancer to access. (Parent/carer)

The importance of age‐appropriate care and professional awareness of the specific needs of young people was highlighted:

If a TYA is treated in a non tya specific clinic, the consultant/haematologists/oncologist etc should be made aware that TYAs are different to adults and should be treated as such. (Patient/former patient)

I had my first chemo on the adult ward and it was awful. Everyone was looking at me with pitying eyes. After that I was introduced to the (name of young person's unit) and it was SO much better. There should be one of these units in every oncology hospital. (Patient/former patient)

Difficulties in accessing specialist services following treatment such as rehabilitation and long‐term effect expertise were also described.

Access to treatment was an issue described by professionals; parents queried the availability of drugs in the UK compared with other countries and young people's access to new drugs and clinical trials. Some parents wanted care to be more holistic and taking into account the young person's lifestyle and factors such as diet. Information about and access to complementary therapies was also mentioned:

What are the possible reasons for adult oncology/haematology consultants being reluctant to refer patients to homeopathic, alternative and supplementary therapies? (Professional)

### 3.6 Long‐term effects and aftercare support

Many participants' comments are related to life after cancer, in particular long‐term effects and aftercare. Some commented on feeling *‘abandoned’* when treatment ended:

After my son completed treatment it felt like we was left to our own devices there doesn't seem to be enough aftercare (Parent/carer)

Young people described living with long‐term effects and needing support:

Are there any groups for individuals who have been left with disabilities after their cancer treatment? Ie So that they can talk to each other or meet up. (Patient/former patient)

Parents raised similar concerns:

Why are the long term effects and resulting depression not discussed more and explained to the patient how they might feel and given tools to deal with these things. (Parent/carer)

Young people asked about on‐going surveillance, frequency of scans/check‐ups, how long they would be monitored for and their necessity. There were also questions about young people's lives after cancer, returning to education or work and achieving life goals:

How can we make sure that the majority of cancer survivors return to society and lead a normal life after treatment? (Professional)

Do people that have cancer at a young age, go on to live normal lives, ie, have children and get married etc? (Patient/former patient)

### 3.7 Family support

Participants, particularly young people, commented on family support needs, the lack of support available and variability in access:

Is there any support for my boyfriend/mum. (Patient/former patient)

Parents wrote about the need for support at all stages of the cancer experience, including at diagnosis, relapse and long‐term survivorship care, and after death:

Being the parent of a young adult with cancer is very lonely. (Parent/carer)

There is an impact on the whole family and this can go on for years. (Parent/carer)

### 3.8 Financial impact

Patients and families highlighted a lack of financial support and issues applying for benefits. Unmet information needs existed about what is available and how to apply for it:

…health and money worries come as a great factor. Filling forms to try and help the families should be easier if the relevant organisations could explain what additional benefits they could be entitled to. (Relative)

What financial help will I get? (Patients/former patient)

Questions were raised about communicating with employers about cancer and taking time off work.

### 3.9 End‐of‐life care

Participants, mainly professionals and parents, offered thoughts and experiences on end‐of‐life care:

It would be nice to meet up with other bereaved parents. (Parent/carer)

Communication about end of life was a concern, for parents, young people and professionals. Professional statements included how best to communicate choices to a young person when treatment is not curative. The need to involve siblings was also raised. Parental concerns included young people's readiness to hear they cannot be cured:

Why do health professionals constantly assume that because a young person seems to be mature, they are mentally ready to hear the news that they have a terminal condition? (Parent/carer)

Some participants wanted more information about illness progression and dying:

How can you make sure their death is painless. (Friend)

More information about the progression of the illness when it's life limiting. (Parent/carer)

Professionals raised concerns whether palliative care service provision meets the needs of young people:

Are there gaps in TYA palliative care provision when compared to adults and children esp 16‐18. (Professional)

I am concerned that hospital and community palliative care teams are asked to see TYA patients but do not have the experience of caring for young patients. (Professional)

### 3.10 Research methods and current research

Queries included why research on young people's cancer remains a low priority. Participants asked about on‐going research on certain topics, for example the long‐term effects of cancer/treatment and where in the world research is being undertaken. There were also queries relating to research methodology and data collection:

How should we collect information about the late‐onset side effects of cancer treatment in TYA? (Professional)

Why is it not automatic that all tumours are sent for research, especially the very rare ones? (Parent/carer)

Some people commented they wanted feedback on research findings:

How do I know about the study I took part in to determine if Hodgkin lymphoma is genetic? With cases in the family, I believe it is. Then, how will I know if my children will have those genes? (Patient/former patient)

## 4 DISCUSSION

What happens after prioritization has become an important focus for the JLA. The release of the top 10 research priority list is not the final step.^9^ As part of that process, the JLA guidebook encourages PSPs to consider the uses of ‘out‐of‐scope’ data. One suggestion is to pass it on to relevant charities to create a frequently asked question list, to be published with the final report or used by them in an awareness campaign.^4^ We choose to work with one of our charitable partners, Young Lives Vs Cancer (previously known as CLIC Sargent), to analyse the ‘out‐of‐scope’ questions and identify an initial list of potential interventions, which will need further refining, developing and testing (Table 1). We reflect here on the ten themes, in relation to published work; further, we describe how three areas of unmet need were identifiable across all of the ten themes: information needs, communication and service provision.

Information needs were by far the largest group of comments. Similar to Bradford et al, most comments related to what would happen after treatment; participants wanted to know about their future and the impact of treatments.^10^ Maintaining an everyday life, pursing life goals, financial independence, support from others and social care were further gaps in information needs. Information may have been shared; however, it might have been given at the wrong time or not communicated in a way to ensure recall or understanding. We previously reported that effective delivery of information is one of the ‘*arts of age‐appropriate car*e’ with young people saying the ‘who’, ‘when’ and ‘how’ were all important for understanding and retention of information.^11^ For participants, information needs prevailed. Unanswered questions existed, questions that related to them or their child; of note were requests for fertility information and lifestyle choices. In some cases, questions revealed limited knowledge of their cancer, treatment and its effects: all of which influence decision making.^12^ What happens when treatment ends was a further knowledge gap. Lea et al refer to an ‘end‐of‐treatment’ transition process, as young people describe being unprepared for the unpredictable and on‐going nature of physical and psychological issues faced when treatment ends.^13^, ^14^ Their call for timely, structured and equitable information resonates with the comments we received and reflects statements about returning to an everyday life and being prepared for what that might mean. The need for a ‘continuing process’ of information is evidenced here; ‘one‐off’ discussions, although timely and relevant, may not have helped to incrementally build a solid platform of knowledge.

Communication was a thread running through many of the themes. Questions were submitted about the delivery of information, and timeliness, and also questions about receiving information and ‘who’ is involved in decision making. Effective communication has at its core patient‐centred communication.^15^ By that we mean communication on the issues are particularly salient to young people. Clearly, for these participants, communication had not always been as effective as they might have wanted it to be, leaving them with unanswered questions about their experiences. Areas of particular need were conversations surrounding poor prognosis and end‐of‐life care, where understanding preferences and the kinds of conversations that are best facilitated with young people are only beginning to be fully examined.^16^, ^17^ Here again, participants' comments point to the need for a ‘long‐term’ communicative relationship, rather than ‘one‐off’ discussions.^18^ Content is seen as one element, albeit an important element of the conversation, timing and facilitation are also essential components of communication.^11^, ^17^ Indeed, tailored conversations, might have helped some of our participants; without that, gaps remain in knowledge, which may have an impact on health‐related decisions for young people and those supporting them.

Service provision, the need for coordinated care, understanding who delivers what and who to go to ask questions featured in participants' questions. Clearly, some had experienced a variable service, raising questions about what was available and what was not; with a call for provision of particular services such as complementary therapies and psychosocial care. Models of care are well established in the UK and contain many of the elements described by Osborn et al, in terms of how care is delivered.^19^ Centralization and coordination between services are key to these models, but for some of these participants, they experienced ‘gaps’, raising concerns about inequalities. Such services are now the focus of evaluation studies where professionals are learning from young people and their family members about how they experience services over time and in a range of settings; not all of which might be described as age‐appropriate.^1^, ^20^, ^21^, ^22^ Inpatient care was only one aspect of participants' concerns, and questions were also raised about availability and coordination of services beyond initial treatment, what might be referred to as support services, for example psychological care^1^ and long‐term follow‐up care.^23^, ^24^ Such services may have been available, but what participants' concerns reveal are gaps, in knowing what they need, what is available and gaps where young people and their families have not been sign posted to services. The need to tailor services, as well as tailor information, and communication is a striking thread throughout.

### 4.1 Implications for practice

When considering the themes together, 'support needs' feature consistently. Supporting the psychological well‐being, social functioning and mental health needs of the young person, while also supporting the family and friend network who are often integral to their care, is the assurance of specialist TYA cancer care. The questions asked were therefore often surprising, particularly those around availability of local services and long‐term late effects. This highlights the need for professionals to continually check‐in with young people and their families about questions they may have and to revisit previous conversations. The care of young people is complex and involves multiple professionals and health and social organizations; ensuring clear pathways and lines of communication between primary care, secondary care, social care, education and employment will support young people and their families through the complexity of the cancer trajectory back to as healthy a life as possible.

### 4.2 Implications for policy and research

Structures and processes to support and deliver TYA specialist care are in place in the UK. We heard here how these can be variably experienced. There is a need for service providers to embrace the concept of age‐appropriate care, to ensure effective communication and delivery of information. The research implications are obvious—we need to encourage research funders to prioritize funding research in rarer cancers and to move away from research strategies dominated by laboratory and clinical research.^25^ A research agenda that reflects the whole cancer experience and is responsive to what patients, carers and professionals need will allow us to generate effective interventions. Neither of these are easy tasks and are compounded by the current climate when many funders are looking to cut their research expenditure in response to the COVID‐19 pandemic.^26^, ^27^ Nevertheless, we must continue to promote the top 10 research questions, and this additional analysis of the out‐of‐scope questions provides further evidence of the need to continue to fund research to inform and evaluate interventions. Table 1 shows the themes alongside subthemes, potential interventions and the related top 10 research priority. Further stakeholder engagement will be required to refine, propose and test interventions derived from the out‐of‐scope questions.

### 4.3 Strengths and limitations

This was a nationwide consultation exercise with patients, carers and professionals having an equal say in the generation of research priorities with the opportunity to submit additional thoughts, experiences and dialogue. We have made full use of the data generated, ensuring the voices of those who did not submit researchable questions are still heard and responded to.

There was under‐representation from minority ethnic groups and male patients within our PSP, as is typical for survey research.^5^, ^28^ Similar to another report, a higher proportion of patients/public submitted ‘out‐of‐scope’ questions compared with professionals, although we do not view this a particular limitation in our PSP as we have used all the ‘out‐of‐scope’ questions.^29^ Despite this, we feel we have captured a wide range of diverse experiences. We are also unable to determine whether information was not delivered to young people and families/carers or whether this information was not retained. However, this does highlight the implications for practice in revisiting information needs throughout the cancer trajectory, including in long‐term follow‐up.

## 5 CONCLUSION

The TYA Cancer PSP generated a top 10 research priority list, and this further analysis of the ‘out‐of‐scope’ questions provides us with an insight into concerns generated by those living with a cancer diagnosis. This is an important outcome, and working with our partners, it will be important to acknowledge and evidence where all the outputs from the PSP have impacted on change, not just the top 10. We have highlighted an approach to responding to ‘out‐of‐scope’ questions and would encourage other PSPs to prioritize this activity too; making sure time and, where needed, funding is made available to undertake this process. What is refered to as ‘post‐PSP action’ (p1) must be planned for, if we are to ensure that there can be learning, as well as new knowledge generated, from respondents who took the time to complete surveys.^9^ We must continue to advocate for specialist care for young people and highlight these concerns and research priorities to funding bodies, policymakers and those involved in service delivery, in order for us to generate an evidence base on which to build effective interventions across the cancer trajectory to improve outcomes for this unique cancer population.

## CONFLICT OF INTEREST

The authors declare that they have no conflict of interest.

## AUTHOR CONTRIBUTIONS

SA, LF, RP, AC, KD, HG, MG, LH, RH, DM, SM, SM, SS, SU, HV, MW, JSW and FG contributed to protocol design, survey refining, data cleaning and refining questions submitted in the initial survey. LF, SA, FG and SU managed the project. SA contributed to survey design. FG, LF and SA coded the survey submissions. SA, LF and RP searched and checked uncertainties. SA and LF managed data entry. All authors were part of the Teenage and Young Adult Cancer Priority Setting Partnership steering group or coordinating team, made substantive contributions to the conduct of the study, overseeing all aspects of the work, and reviewed and approved the final version of this manuscript.

## ETHICAL APPROVAL

Ethical approvals are not required for JLA partnerships as per JLA and National Health Services Patient Safety Agency National Research Ethics Service Guidance (The Health Research Authority/INVOLVE). All procedures performed in studies involving human participants were in accordance with the ethical standards of the institutional and national research committee and with the 1964 Helsinki declaration and its later amendments or comparable ethical standards.

## Supporting information

App S1

## Data Availability

The data that support the findings of this study are available from the corresponding author upon reasonable request.
