# Supplementary material for: Reporting the whole story: Analysis of the ‘out‐of‐scope’ questions from the James Lind Alliance Teenage and Young Adult Cancer Priority Setting Partnership Survey
Source: Health Expect. 2021 Jul 10;24(5):1593–606. doi: 10.1111/hex.13276 (PMC8483195; doi:10.1111/hex.13276)
Supplement: Supplementary file 1 — App S1 [file HEX-24-1593-s001.docx]

**Supplementary material 1 Details of respondents who submitted out of scope questions or comments (n=191)**

|  | **Response** | **Patients/former patients (n=68)** | **Parents/relatives/friends/partners (n=81)** | **Professionals**  **(n=42)** |
| --- | --- | --- | --- | --- |
| **Gender** | Female | 58 (85%) | 64 (79%) | 28 (67%) |
|  | Male | 10 (15%) | 15 (19%) | 14 (33%) |
|  | Other | 0 (0%) | 1 (1%) | 0 (0%) |
|  | Prefer not to answer | 0 (0%) | 1 (1%) | 0 (0%) |
| **Ethnic group** | White | 63 (93%) | 78 (96%) | 38 (90%) |
|  | Asian or Asian British | 3 (4%) | 1 (1%) | 1 (2%) |
|  | Black African, Black Caribbean or Black British | 1 (1%) | 0 (0%) | 1 (2%) |
|  | Mixed/multiple ethnic groups | 1 (1%) | 0 (0%) | 1 (2%) |
|  | Prefer not to answer | 0 (0%) | 1 (1%) | 1 (2%) |
|  | Missing data | 0 (0%) | 1 (1%) | 0 (0%) |
| **Age (years)** | 13-15 | 2 (3%) | 0 (0%) | n/a |
|  | 16-18 | 6 (9%) | 3 (4%) | n/a |
|  | 19-24 | 31 (46%) | 3 (4%) | 0 (0%) |
|  | 25-34 | 25 (37%) | 4 (5%) | 4 (10%) |
|  | 35-44 | 3 (4%) | 14 (17%) | 13 (31%) |
|  | 45-54 | 1 (1%) | 36 (44%) | 16 (38%) |
|  | 55-64 | 0 (0%) | 18 (22%) | 8 (19%) |
|  | 65+ | 0 (0%) | 3 (4%) | 1 (2%) |
| **Country of residence (patients/former patients/parents/relatives/friends/ partners)**  **Country of work (professionals)** | England | 57 (84%) | 60 (74%) | 32 (76%) |
|  | Scotland | 7 (10%) | 11 (14%) | 5 (12%) |
|  | Wales | 3 (4%) | 5 (6%) | 3 (7%) |
|  | Northern Ireland | 1 (1%) | 2 (2%) | 0 (0%) |
|  | Other | 0 (0%) | 3 (4%) | 2 (5%) |
| **Cancer first diagnosed with** | Hodgkin's disease/lymphoma | 17 (25%) | 13 (16%) | n/a |
|  | Leukaemia | 12 (18%) | 14 (17%) | n/a |
|  | Bone tumour (sarcoma) | 5 (7%) | 8 (10%) | n/a |
|  | Non-Hodgkin’s Lymphoma | 6 (9%) | 12 (15%) | n/a |
|  | Brain or spinal cord | 5 (7%) | 13 (16%) | n/a |
|  | Soft tissue tumour (sarcoma) | 4 (6%) | 8 (10%) | n/a |
|  | Ovarian | 3 (4%) | 0 (0%) | n/a |
|  | Carcinoma | 0 (0%) | 1 (1%) | n/a |
|  | Thyroid | 1 (1%) | 1 (1%) | n/a |
|  | Testicular | 4 (6%) | 1 (1%) | n/a |
|  | Melanoma (skin cancer) | 3 (4%) | 0 (0%) | n/a |
|  | Colorectal (bowel cancer) | 1 (1%) | 2 (2%) | n/a |
|  | Breast | 1 (1%) | 0 (0%) | n/a |
|  | Cervical | 1 (1%) | 0 (0%) | n/a |
|  | Other | 5 (7%) | 5 (6%) | n/a |
|  | Not sure | 0 (0%) | 3 (4%) | n/a |
| **Current situation*** | Survivor/Follow-up care | 46 (68%) | 24 (30%) | n/a |
|  | End of treatment | 6 (9%) | 7 (9%) | n/a |
|  | On treatment | 4(6%) | 18 (22%) | n/a |
|  | Relapsed | 2 (3%) | 0 (0%) | n/a |
|  | Palliative and end of life care | 0 (0%) | 1 (1%) |  |
|  | Deceased | n/a | 26 (32%) | n/a |
|  | End of treatment, Survivor/Follow-up care | 9 (13%) | 1 (1%) | n/a |
|  | On treatment, Relapsed | 1 (1%) | 2 (2%) | n/a |
|  | End of treatment, Relapsed | 0 (0%) | 1 (1%) | n/a |
|  | Not sure | 0 (0%) | 1 (1%) | n/a |

*Participants could select more than one response
